# Application of Sonotriboluminescence to Determine Arene Molecules in Hydrocarbons

**DOI:** 10.3390/molecules28237932

**Published:** 2023-12-04

**Authors:** Adis A. Tukhbatullin, Nadezhda A. Panova, Dim I. Galimov, Bulat M. Gareev, Alina A. Tukhbatullina, Kristina S. Vasilyuk, Glyus L. Sharipov

**Affiliations:** High-Energy Chemistry and Catalysis Laboratory, Institute of Petrochemistry and Catalysis UFRC RAS, 141, Oktyabrya Prosp., 450075 Ufa, Russia; naduasha.panova99@mail.ru (N.A.P.); galimovdi@mail.ru (D.I.G.); gareev-bulat@yandex.ru (B.M.G.); kalieva.alina@rambler.ru (A.A.T.);

**Keywords:** ultrasound, aromatic molecules, lanthanide salts, triboluminescence

## Abstract

The sonotriboluminescence of suspensions of terbium(III) and europium(III) sulfates in decane without and in the presence of benzene, toluene and *p*-xylene was studied. The choice of crystals of these lanthanides is due to the fact that they have intense luminescence during mechanical action, and also do not dissolve in hydrocarbon solvents. During ultrasonic exposure to suspensions in pure alkanes, bands of Ln^3+^ ions and N_2_ in the UV region are recorded in the luminescence spectrum. When aromatic hydrocarbon molecules are added, bands of benzene, toluene and *p*-xylene molecules, coinciding with their photoluminescence spectra, are recorded in the sonotriboluminescence spectra in the UV region. The high sensitivity of the luminescence of suspensions to arene additives made it possible to obtain the dependence of the characteristic fluorescence of arene molecules in the sonotriboluminescence spectra on their concentration in suspensions. The limits of detection of benzene, toluene and *p*-xylene in the composition of this suspension were established. The lower limits of detection from the sonotriboluminescence spectra for xylene, toluene and benzene are 0.1, 3 and 50 ppmv, respectively. Fluorescence bands of these molecules were also recorded in the sonotriboluminescence spectra of suspensions in commercial dodecane and heptane with additives of commercial gasoline (up to 1%). The results obtained can be used for luminescent detection of aromatic compounds in saturated hydrocarbons.

## 1. Introduction

Sonotriboluminescence (STL), a sonogenerated emission that occurs during the destruction of crystals in suspensions, is of not only fundamental (study of the mechanisms of conversion of mechanical energy into light) but also of practical interest for the development of new luminescent applications, especially in the field of analytical chemistry. Today, one of the most promising classes of sono- and triboluminophores are lanthanide compounds with intense luminescence in the near-UV (Ce^3+^, Dy^3+^), blue (Eu^2+^), green (Tb^3+^), red (Eu^3+^, Sm^3+^) and IR (Yb^3+^) regions of the electromagnetic spectrum. Previously, we reported the registration of multiemitter sonotriboluminescence that occurs during sonication of suspensions of terbium sulfate in certain hydrocarbons [1,2,3]. It has been established that the intensity of STL of suspensions containing Tb_2_(SO_4_)_3_ crystals significantly exceeds the intensity of sonoluminescence (SL) of a homogeneous solution of Tb_2_(SO_4_)_3_. Moreover, the STL spectra, in addition to narrow emission of Tb^3+^, contain intense fluorescence bands of aromatic hydrocarbons (benzene, toluene, *p*-xylene) in the region of 280–290 nm [1]. The multibubble SL spectrum of benzene, toluene and xylene is recorded only in the form of a wide structureless continuum [2]. Such differences in the spectral brightness characteristics of STL and SL are due to different mechanisms for excitation of luminescence during ultrasonic treatment of liquids and suspensions. SL occurs due to the collapse of cavitation bubbles as a result of their thermal heating reaching several thousand Kelvin [4,5,6,7]. STL is mainly caused by the occurrence of electrical discharges [8,9,10,11,12,13] during the collision and subsequent destruction of suspension crystals moving under the action of cavitation shock waves. In this regard, during ultrasonic treatment of suspensions, the STL spectrum contains more clearly defined emission bands than during SL of true solutions [2,8]. The mechanism of the phenomena described above still remains poorly understood despite numerous studies in the field of ultrasound technology. Therefore, the development of research in the field of ultrasound-initiated mechanochemical transformations and luminescence of molecules and ions in suspensions can become the basis for the subsequent creation of chemical–technological processes of sonotribolysis with the possibility of luminescent control over them.

Here, for the first time, we present the results of a comparative study of ultrasonic influence on suspensions of Eu_2_(SO_4_)_3_∙8H_2_O and Tb_2_(SO_4_)_3_∙8H_2_O crystals in alkanes with the addition of different concentrations of arene molecules and commercial hydrocarbons to these liquids to identify patterns of acoustic influence, concentration dependences of luminescence intensities of arenes and the composition of sonotriboluminescence emitters.

## 2. Results and Discussion

**Figure 1:**
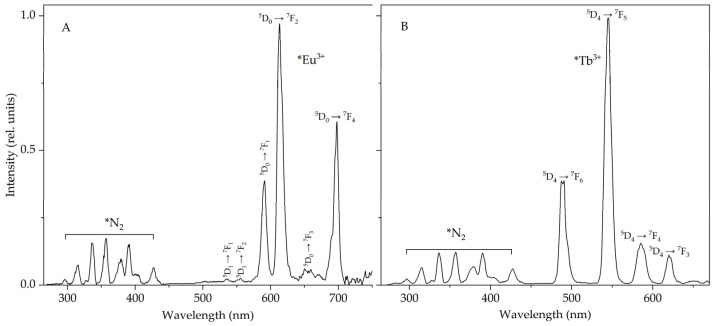
Sonotriboluminescence spectra of suspensions of europium (**A**) and terbium (**B**) sulfate in octane and decane, respectively, in an air atmosphere. Δλ = 5 nm.

During ultrasonic exposure of suspensions of terbium and europium sulfate crystals in higher n-alkanes (from decane to hexadecane [8,14,15,16]), in addition to the emission of the Eu^3+^ and Tb^3+^ ions, N_2_ lines are also recorded in the STL spectra (Figure 1). The molecular nitrogen lines are observed mainly in the UV region [17,18]. The fluorescence bands of some polyaromatic hydrocarbons (PAHs) are also located in this region, and N_2_ luminescence may interfere with the detection of PAHs at low concentrations with unambiguous identification of the fluorescence bands of these hydrocarbons. Therefore, when using the sonotriboluminescent method for detecting PAHs, it is advisable to use, for example, heptane, octane and nonane, in which the N_2_ lines do not appear so effectively. As shown earlier [1,16], this is due to the high saturated vapor pressure in these liquids.

**Figure 2:**
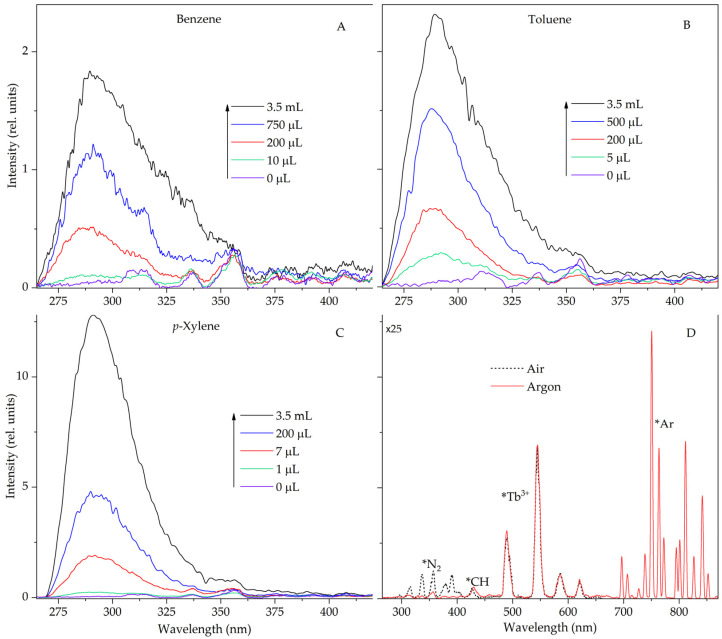
Sonotriboluminescence spectra of suspensions of terbium sulfate in decane (7 mL) in the region of 265–420 nm with different additions of benzene (**A**), toluene (**B**) and *p*-xylene (**C**) in an argon atmosphere (**D**). Sonotriboluminescence spectra of suspensions of terbium sulfate in decane in the region of 265–850 nm in an air (dot line) and argon (solid line) atmosphere. Δλ = 5 nm.

In this work, alkanes are considered when benzene, toluene and *p*-xylene were added to them. In the STL spectra of suspensions containing aromatic hydrocarbons, the luminescence bands of arenas are located below 300 nm. N_2_ lines are not observed in this region and do not create difficulties for recording the luminescence of simple arene molecules. In addition, to displace residual or dissolved nitrogen, the suspension is additionally saturated with argon. Figure 2 shows the STL spectra of suspensions of terbium(III) sulfate in decane at different concentrations of benzene, toluene and *p*-xylene. Intense fluorescence bands of these molecules with main maxima at 285 nm (benzene), 288 nm (toluene) and 292 nm (*p*-xylene), caused by singlet–singlet transitions, are recorded in the spectrum [19,20]. As can be seen from Figure 2, the luminescence of arene molecules in the STL spectrum is easily recorded even at low concentrations in the system.

Recall that, in contrast to sonoluminescence (collisional excitation of emitters inside cavitation bubbles), the mechanism of STL excitation of crystal suspensions is close to the mechanism of triboluminescence, namely, it is associated with electrification and the occurrence of discharges during friction and destruction of crystals [10,11,21,22,23]. Obviously, the acoustic impact and the resulting cavitation shock waves in suspensions lead to high-speed collisions of crystals in the reactor with their subsequent destruction/grinding. This leads to an increase in the active surface and the number of defects in crystals, accompanied by electrification and generation of low-energy electrons. In addition to excitation of the main emitters of the STL spectrum (Ln^3+^ and N_2_), the generated electrons enter the volume of the liquid base of the suspension, and produce radiolysis, exciting the luminescence of aromatic compounds when they are present in alkanes.

**Figure 3:**
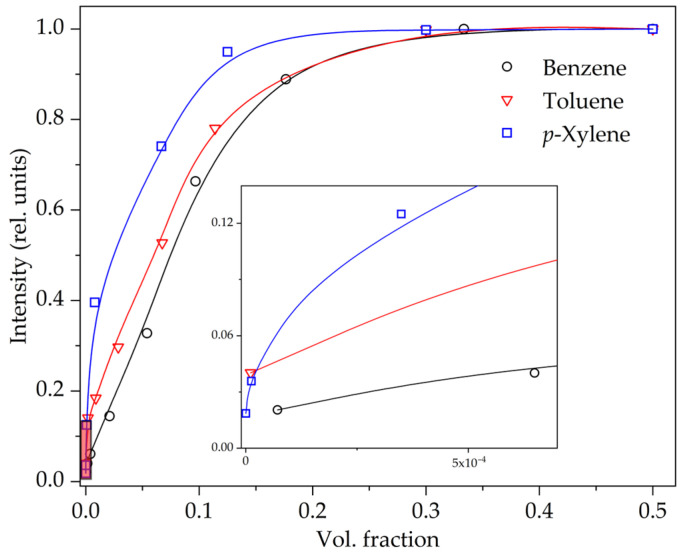
Dependence of the luminescence intensity of arene molecules on the content of benzene, toluene and *p*-xylene during STL of a suspension of terbium(III) sulfate in decane. The inset shows the area with low values.

It should be noted that in a mixture of arenes, it is quite difficult to determine the individual content of benzene, toluene or *p*-xylene from fluorescence spectra. The main maxima of these aromatic hydrocarbons are located very close together, and the bands also have a vibrational structure. When these spectra are superimposed on each other, it is difficult to deconvolute and assign fluorescence bands to one or another arena. However, based on the results obtained individually for each arena on the STL spectra, curves of the dependence of the luminescence intensity of aromatic hydrocarbon molecules on their content in decane were constructed (Figure 3). The detection limit of benzene, toluene and *p*-xylene from the STL spectrum of the suspension in decane is 50 µL·L^−1^, 3 µL·L^−1^ and ~0.1 µL·L^−1^, respectively.

**Figure 4:**
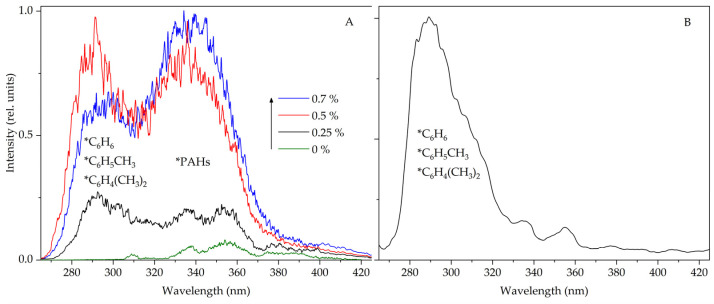
Sonotriboluminescence spectra of a suspension of terbium(III) sulfate in heptane containing commercial gasoline (**A**) and a suspension of europium(III) sulfate in commercial dodecane (**B**). Sonotriboluminescence spectra were recorded in an argon atmosphere, Δλ = 6 nm.

The noticeable influence of the studied arenes on the STL spectra of suspensions can make it possible to use this type of luminescence to determine the molecules of aromatic hydrocarbons in the composition of various n-alkanes and petroleum products. For example, STL of a suspension in commercial gasoline was previously reported [3], caused by the emission of PAHs included in gasoline, the concentration of which is not high (<1%), and according to the literature, some of them (pyrene, anthracene, fluorene, benzo(a)pyrene) can reach up to 6 mg·L^−1^ [24,25]. Note that the content of simple aromatic hydrocarbons in commercial gasoline can reach 35% [26]. However, as shown in [3], at such concentrations (>5%) of gasoline in heptane, the emission bands of benzene, toluene and xylene are almost completely suppressed, and then in the STL spectra, mainly emission bands of PAHs with high luminescence quantum yields are observed. The increase in the emission intensity of PAHs with the subsequent suppression of the emission of simple arenes is explained by the highly efficient transfer of excitation energy from benzene, toluene and *p*-xylene molecules to PAHs [3]. In this regard, to record the luminescence of molecules of simple arenes, STL experiments were carried out on suspensions containing a small amount of gasoline in heptane. Figure 4A shows the STL spectra of suspensions containing from 0.1 to 0.7% commercial gasoline. As can be seen, the most effective bands of benzene, toluene and *p*-xylene molecules with a maximum of ~290 nm are recorded at concentrations less than 1%. Increasing the concentration of gasoline increases the intensity of PAHs in the emission spectrum.

Also, the capabilities of the sonotriboluminescent method for determining aromatic hydrocarbon molecules in different solvents have been tested on commercial dodecane (Figure 4B) of a pure-for-synthesis grade (the content of the main chemical compound is about 99%). No noticeable peaks are observed in the HPLC chromatogram except for the peak of dodecane. At the same time, in the STL spectrum of a suspension of europium(III) sulfate in commercial dodecane, an intense band with a maximum of 290 nm is recorded in the UV region, which indicates the presence of simple arenes.

In the future, we plan to continue research in this direction, expand the range of analyzed aromatic hydrocarbons and determine the possibilities of analyzing arenes in mixtures, as well as to use sonotribolysis and sonotriboluminescence of suspensions during the processing of petroleum products for spectral–luminescent monitoring of this process. In particular, it can be noted that *p*-xylene, unlike benzene and toluene, has a higher quantum yield of luminescence and, therefore, sonotriboluminescence is a promising approach to the development on its basis of analytical methods for the determination of *p*-xylene in a mixture of aromatic hydrocarbons.

## 3. Materials and Methods

Heptane and decane (≥99.99%), benzene, toluene, *p*-xylene (≥99%) without additional purification, commercial dodecane (99%) and commercial gasoline (octane number 92) were used in the work. Suspensions were prepared by adding Eu_2_(SO_4_)_3_∙8H_2_O and Tb_2_(SO_4_)_3_∙8H_2_O crystals (99.99%, Lanhit) to liquids. Sonotriboluminescence was excited in 7 mL of a solvent containing 250 mg of crystals in a thermostated stainless steel reactor with a quartz window at the bottom [1,2]. Ultrasonic exposure was carried out using an ultrasonic dispersant generator UZDN-2T (22 kHz), with a submersible titanium waveguide with a flat end (d = 10 mm). The end of the waveguide was installed at a distance of ~1 mm from the bottom of the cuvette during STL recording. The suspensions were saturated with argon (99.999%) by bubbling at a rate of up to 10 mL·s^−1^. The temperature in the cuvette during recording of STL of suspensions was maintained using a circulation thermostat LT-105a (LOIP, St. Petersburg, Russia). Sonotriboluminescence spectra were recorded using a Zolix OmniFluo-900 spectrofluorimeter (Detector TE-cooled PMT).

All measurements were carried out in a series of similar experiments 3–5 times. The average relative measurement error was no more than 5%.

The identification of dodecane was carried out on a Chromatec-Crystal-5000 gas chromatograph with an Agilent DB-1 50 m × 0.25 mm × 0.25 µm capillary column (programmed heating from 100 to 270 °C at a rate of 8 °C min, helium carrier gas). The chromatograph was attached by interface to the computer where the data were acquired with the program Chromatec Analitic 3.0.0.2.

## 4. Conclusions

Thus, the sonotriboluminescence of suspensions of lanthanide salts in heptane and decane with the addition of arene molecules and commercial gasoline, as well as a suspension in commercial dodecane, was studied. The dependences of the intensities of characteristic fluorescence were obtained and limits of detection of benzene, toluene and *p*-xylene molecules in the composition of decane were established. The lower limits of detection from the sonotriboluminescence spectra for xylene, toluene and benzene are 0.1, 3 and 50 ppmv, respectively. Fluorescence bands of these molecules were also recorded in the sonotriboluminescence spectra of suspensions in commercial dodecane and heptane with additives of commercial gasoline (up to 1%). The results obtained can be used for luminescent detection of aromatic compounds in saturated hydrocarbons.

## Data Availability

Data are contained within the article.
